# Sericin’s Potential in Osteoporosis Management: The Roles of L-Serine and D-Serine in Bone Metabolism Regulation

**DOI:** 10.3390/nu17030574

**Published:** 2025-02-04

**Authors:** Jwa-Young Kim, Xiangguo Che, Shihyun Kim, Jongho Choi, Joon Ha Lee, Ji-Hae Lee, HaeYong Kweon, Suk Keun Lee, Je-Yong Choi, Seong-Gon Kim

**Affiliations:** 1 Department of Oral and Maxillofacial Surgery, Hallym University Kangnam Sacred Heart Hospital, Hallym University Medical Center, 1 Singil-ro, Yeongdeungpo-gu, Seoul 07441, Republic of Korea; jwayoung@hanmail.net; 2 Department of Biochemistry and Cell Biology, Cell and Matrix Research Institute, School of Medicine, Kyungpook National University, Daegu 41944, Republic of Korea; xiangguo0622@naver.com; 3 Department of Oral Pathology, College of Dentistry, Gangneung-Wonju National University, 7 Jukheon-gil, Gangneung-si 25457, Gangwon-do, Republic of Korea; shihyun7372@hanmail.net (S.K.); jhchoi@gwnu.ac.kr (J.C.); 4 Industrial Insect and Sericulture Division, National Institute of Agricultural Science, Rural Development Administration, Wanju 55365, Republic of Korea; coover@korea.kr (J.H.L.); jihae@korea.kr (J.-H.L.); hykweon@korea.kr (H.K.); 5 Institution of Hydrogen Magnetic Reaction Gene Regulation, Daejeon 34140, Republic of Korea; sukkeunlee@hanmail.net; 6 Department of Oral and Maxillofacial Surgery, College of Dentistry, Gangneung-Wonju National University, 7 Jukheon-gil, Gangneung-si 25457, Republic of Korea

**Keywords:** sericin, L-serine, D-serine, osteoporosis, bone metabolism, osteoblast, osteoclast

## Abstract

Background: Osteoporosis is a bone remodeling disease characterized by an imbalance between bone formation and resorption, leading to bone fragility. Current treatments focus on bone resorption suppression but often have adverse effects. This study aimed to explore the potential of sericin, a silkworm-derived protein, as a dual-action therapeutic agent that enhances bone formation through its component L-serine and inhibits bone resorption via D-serine, which is derived from L-serine by the action of serine racemase. Methods: Cellular experiments were conducted to evaluate the effects of L-serine on osteoblast differentiation and D-serine on osteoclast inhibition. Serum levels of D-serine were measured following sericin administration in an osteoporosis animal model. μ-CT analysis assessed trabecular and cortical bone quality, and bone-related protein expression was analyzed using immunoprecipitation-based high-performance liquid chromatography (IP-HPLC). Results: L-serine significantly upregulated osteogenic markers, including alkaline phosphatase (ALP), Runx2, osterix, and Col1a1, in osteoblasts (*p* < 0.05). D-serine inhibited osteoclast activation by suppressing cathepsin K expression (*p* < 0.001). Sericin feeding elevated serum D-serine levels (*p* < 0.001) and upregulated bone-related proteins such as BMP-2, osterix, and Runx2. Micro-computed tomography (μ-CT) analysis revealed significant improvements in trabecular bone parameters in the OVX-sericin group, including increased trabecular bone volume (Tb.BV/TV; *p* < 0.05) and reduced trabecular separation (Tb.Sp; *p* < 0.05), compared to the OVX and OVX-amino acid groups. Cortical bone parameters, including cortical bone volume (Ct.BV/TV) and cortical area (Ct.Ar), did not significantly differ among OVX groups, but all were lower than in the sham group (*p* < 0.05). Conclusions: This study demonstrates that sericin modulates bone metabolism by enhancing osteoblast activity through L-serine and inhibiting osteoclastogenesis via D-serine. Sericin supplementation improved trabecular bone mass in an osteoporosis model, highlighting its potential for bone health.

## 1. Introduction

Osteoporosis is a common disease characterized by the progressive loss of bone mass due to an imbalance between bone formation and resorption, leading to an increased risk of fractures [1,2]. While primarily affecting postmenopausal women, osteoporosis also poses significant health risks to men [3]. The complications associated with osteoporosis, particularly fractures, contribute to increased mortality and reduced quality of life [4]. Therefore, anti-resorptive drugs are essential for high-risk groups to prevent bone loss and reduce fracture risk [5].

Current treatment strategies focus on anti-resorptive drugs such as bisphosphonates and receptor activator of nuclear factor kappa-B ligand (RANKL) inhibitors, which aim to suppress bone resorption and prevent fractures [6,7]. However, these therapies are often associated with adverse effects, including medication-related osteonecrosis of the jaw (MRONJ), limiting their long-term use [8]. In addition to pharmacological interventions, calcium-based functional foods have been widely used as supplementary regimens for osteoporosis patients [9]. However, excessive calcium is excreted via the kidneys and does not contribute to increased bone mass [10]. Moreover, there is an upper limit to the daily calcium intake, limiting its efficacy as a preventive measure [10]. These challenges underscore the need for alternative therapeutic approaches with improved safety profiles and mechanisms that simultaneously address bone formation and resorption.

Sericin, a protein derived from *Bombyx mori* (silkworm), has emerged as a promising candidate in bone metabolism research due to its unique composition [11]. Approximately 30% of sericin’s structure consists of L-serine, significantly higher than other proteins [12]. When administered orally, sericin elevates systemic levels of L-serine, which can be converted to D-serine by serine racemase in cells [13]. Prior studies suggest distinct roles for these amino acids: L-serine promotes osteoblast differentiation and bone formation, while D-serine inhibits osteoclast activity and bone resorption [14,15]. However, the precise mechanisms underlying these effects remain incompletely understood, particularly regarding their combined influence on systemic bone metabolism. While sericin-based interventions have demonstrated improvements in bone mineral density (BMD) and quality in osteoporosis models [16], key questions remain unresolved. For instance, the impact of sericin-derived D-serine on bone metabolism, as well as comparisons between sericin and other amino acid-based therapies, have yet to be comprehensively explored.

This study aims to address these gaps by investigating the distinct roles of sericin’s bioactive components—L-serine and D-serine—in bone metabolism. Specifically, we evaluate their effects on osteoblast and osteoclast activities, the systemic impact of sericin feeding on D-serine levels, and changes in trabecular and cortical bone quality in an osteoporosis animal model. By elucidating the mechanisms underlying sericin’s dual action, this study provides a foundation for its potential development as a therapeutic agent for osteoporosis management.

## 2. Materials and Methods

### 2.1. Cellular Experiment

In the cellular experiments, L-serine was tested at concentrations of 0.1, 1, and 10 mM, while D-serine was tested at concentrations of 10, 50, and 100 μM. The concentration range for D-serine was selected based on a previous study demonstrating its inhibitory effects on osteoclast activity within this range [14]. L-serine concentrations were chosen to reflect the expected transformation of L-serine to D-serine in biological systems, as approximately 8.55% of ingested L-serine is converted to D-serine by serine racemase [17]. Therefore, the tested L-serine concentrations were set at least 10 times higher than those for D-serine to account for this conversion and ensure the biologically relevant testing of its osteogenic effects.

MC3T3E1 cell was purchased from the Korean Cell Line Bank (CKLB; Seoul, Republic of Korea). They were seeded in 6-well plate at a density of 3 × 10^5^ cell/well in a α-MEM containing 1% antibiotics and 10% fetal bovine serum (FBS; Gibco; Thermo Fisher Scientific, Inc., Waltham, MA, USA), treated with various concentrations (0, 0.1, 1, 10 mM) of L-serine. Total RNA was isolated from cells after 8 and 24 h of treatment using an easy-BLUE Total RNA Extraction Kit (iNtRON Biotechnology, Seongnamsi, Gyeonggi-do, Republic of Korea) according to the manufacturer’s instructions. Complementary DNA (cDNA) was synthesized from 2 μg of total RNA using SuperScript II Reverse Transcriptase (Invitrogen, Carlsbad, CA, USA). Quantitative real-time PCR (qRT-PCR) was conducted using the Power SYBR Green Mas-ter Mix (Applied Biosystems, Foster, CA, USA). The PCR reaction system consisted of 2 μL of 40× diluted cDNA, 5 μL of SYBR^®^ Premix Ex Taq (Takara Bio Inc., Kusatsu, Japan), and 0.25 μM of each primer pair, in a total reaction volume of 10 μL. The thermal cycling conditions were as follows: initial denaturation at 95 °C for 10 min, followed by 40 cycles of 15-s denaturation at 95 °C and then 60 s annealing and extension at 60 °C. The qRT-PCR results were analyzed using the ΔΔCt method to determine the relative expression of target genes, normalized to a *GAPDH* gene and compared to a control sample. The primer design is shown in Table A1.

The RAW 264.7 cell line was sourced from CKLB. These cells were maintained in Dulbecco’s modified eagle medium (DMEM; Invitrogen; Thermo Fisher Scientific, Inc., Waltham, MA, USA) supplemented with 10% FBS and 1% penicillin–streptomycin (P/S; Gibco; Thermo Fisher Scientific, Inc.). Cells were cultured in a humidified incubator at 37 °C with 5% CO_2_. To prevent differentiation into osteoclasts, RAW264.7 cells were pretreated with 175 μM D-serine in DMEM for 24 h. For osteoclast differentiation, cells were treated with 50 ng/mL recombinant human-soluble RANK Ligand (RANKL; Oriental Yeast, Tokyo, Japan) and 20 ng/mL recombinant mouse macrophage colony-stimulating factor (M-CSF; R&D Systems, Minneapolis, MN, USA) in α-MEM medium containing 1% P/S for 5 days, with the induction medium being replaced every 2 days. The change in cathepsin K expression levels was evaluated 5 days after sericin administration and compared to cells without D-serine pretreatment.

### 2.2. Western Blot Analysess

RAW 264.7 cells were seeded at a density of 8 × 10^3^ cells per 60 mm culture plate and incubated overnight at 37 °C in a humidified atmosphere with 5% CO_2_. Cells were pretreated with D-serine for 24 h, after which the medium was changed to α-MEM containing RANKL and M-CSF to induce osteoclast differentiation for 5 days. Protein extracts were collected using Laemmli buffer supplemented with protease inhibitor (Roche Diagnostics, Basel, Switzerland) and phosphatase inhibitor cocktail (Roche Diagnostics). After heat denaturation, the proteins were separated on 8–15% acrylamide gels by electrophoresis at 95 V for 3 h. The separated proteins were transferred onto 0.45 µm nitrocellulose membranes (Bio-Rad Laboratories, Inc., Hercules, CA, USA) at 20 V overnight. The membranes were blocked and then incubated with primary antibodies at a 1:1000 dilution. The primary antibodies used were cathepsin K (cat. no. sc-48353; Santa Cruz Biotechnology, Santa Cruz, CA, USA) at 1:1000 dilution in BSA and GAPDH (cat. no. LF-PA0212; AbFrontier, Seoul, Republic of Korea) at 1:3000 dilution in BSA. The blots were visualized using a FUSION Solo S imaging system (Vilber Lourmat, Collégien, France), and the intensity of the blots was quantified as fold-change using ImageJ software (version 1.53a; National Institutes of Health, Bethesda, MA, USA).

### 2.3. Tartrate-Resistant Acid Phosphatase (TRAP) Activity Assay

For RAW264.7 cells, 3 × 10^3^ cells were seeded on coverslips in a 6-well plate and incubated overnight at 37 °C in a 5% CO_2_ atmosphere. Cells were either pretreated with or without D-serine, and differentiated into osteoclasts in the presence of RANKL and M-CSF for 5 days. After differentiation, the cells were fixed with 4% paraformaldehyde, permeabilized with 0.5% Triton X-100 (Sigma-Aldrich, St. Louis, MO, USA) for 10 min at room temperature, and washed three times with PBS for 3 min. TRAP staining was performed using a TRAP staining kit (Solarbio, Beijing, China) according to the manufacturer’s instructions. The samples were fixed with TRAP fixative at 4 °C for 3 min, washed with distilled water, and incubated with TRAP Incubation Solution. After 60 min of incubation at 37 °C in the dark, the samples were washed three times with distilled water for 3 min, counterstained with Hematoxylin Solution (Sigma-Aldrich, St. Louis, MO, USA) for 5 min at room temperature, and mounted using a mounting medium. Images were obtained using an upright microscope (BX53; Olympus Corporation, Tokyo, Japan) at a magnification of ×10.

### 2.4. Animal Experiment

In this study, we utilized ovariectomized rats as a model for osteoporosis due to their established validity in simulating postmenopausal osteoporosis, which is a primary cause of bone loss and fragility in humans. OVX in rats induces hormonal changes like those seen in postmenopausal women, resulting in decreased bone density, and altered bone metabolism. This model is widely recognized for its relevance in evaluating potential osteoporosis therapies, allowing for translational insights into the efficacy of bone regeneration treatments [16]. All procedures were performed in accordance with the guidelines for laboratory animal care and were approved by the Gangneung-Wonju National University for animal research (GWNU-2024-4 approved at 23 April 2024 and GWNU-2024-14-1 approved at 6 December 2024). Six-week-old Crl:CD (Sprague–Dawley) specific pathogen-free (SPF)/VAF outbred rats (Orientbio Inc., Sungnam, Republic of Korea) were used in this study. Thirty rats (2–3 rats per cage) were housed under a 12 h light/12 h dark cycle in a controlled environment at 20–22 °C and 40% humidity for one week for acclimation prior to experimentation. The rats had free access to food and water, and were all fed a controlled semisynthetic diet according to a classical recommendation (74% carbohydrates from soybean vegetable oil, 14% protein from casein, supplemented with a standard vitamin and mineral mix).

All rats underwent OVX, and after a 3-month recovery period, two rats died, leaving the remaining rats to be divided into three groups: OVX (*n* = 10), OVX-amino acid (25 mg/kg, *n* = 10), and OVX-sericin (25 mg/kg, *n* = 8). The OVX group received sericin-free saline as a control, while the OVX-amino acid and OVX-sericin groups were orally administered the respective treatments by mixing the amino acid mixture or sericin into their drinking water for 8 weeks, starting 4 weeks post-OVX. The amino acid mixture was purchased from Sigma-Aldrich (CAT#: AAS18, Sigma-Aldrich, St. Louis, MO, USA). This mixture contains the following amino acids, each at a concentration of 2.50 μmol/mL (±4%): L-alanine, L-arginine, L-aspartic acid, L-glutamic acid, L-histidine, L-isoleucine, L-leucine, L-lysine, L-methionine, L-phenylalanine, L-proline, L-serine, L-threonine, L-tyrosine, L-valine, and glycine. Additionally, it contained L-cystine at a concentration of 1.25 μmol/mL (±4%). L-serine, a key component in the mixture, constituted 10% of the total amino acid content, providing a basis for comparison with the sericin group. According to the datasheet, 25 mg of the mixture contained approximately 1.39 mg of L-serine. At 21 weeks of age, after 8 weeks of oral treatment, all rats were euthanized using CO_2_ gas followed by cervical dislocation. Femora were collected for further analysis. For the comparative analysis of μ-CT results, data from a sham operation group (*n* = 8) obtained from Prof. Ji-Hyeon Oh (Gangneung-Wonju National University) were included.

### 2.5. D-Serine Quantification in Serum Samples

Serum samples should be clarified by centrifugation at 10,000× *g* for 5 min to reduce turbidity and remove insoluble material. After centrifugation, the supernatant was carefully transferred to a new microfuge tube. This clarified serum was used for further analysis. To eliminate potential interference from common metabolites found in serum, the sample was pretreated with Sample Cleanup Mix and deproteinized. For each serum sample, Sample Cleanup Mix was added at a 1:25 ratio. The mixture was incubated at 37 °C for 15 min. Following incubation, the treated serum was transferred to a 10 kDa MWCO Spin Column. The samples were centrifuged at 10,000× *g* for 10 min to separate the filtrate. The filtrate represented the deproteinized serum, which was stored at −20 °C for future experiments for up to 2 months.

Of the pretreated, filtered serum 5 µL was added to the desired wells in a black, flat-bottom 96-well plate. For accurate quantification, at least three parallel wells were prepared for each serum sample: one for the determination of D-serine only, one for the determination of total serine (including both D- and L-isomers), and one as a sample background control. The volume of each well was adjusted to 60 μL using a serine assay buffer. The 96-well plate containing the prepared serum samples was preincubated at 37 °C for 10 min. During the preincubation period, the reaction mixes for the D-Serine Only, Total Serine, and Sample Background Control wells were prepared according to the specific requirements for each type. The plate was incubated at 37 °C for 60 min, ensuring that it was protected from light to prevent any photobleaching or unwanted reactions. After incubation, the fluorescence of all sample, background, and standard curve wells was measured using a fluorescence plate reader set to an excitation wavelength of 535 nm and an emission wavelength of 587 nm in the endpoint mode.

### 2.6. IP-HPLC

The protein samples were analyzed by IP-HPLC as follows. Protein A/G agarose columns were separately preincubated with 1 μg of each antibody. The details of the antibodies used are listed in Table A2. The supernatant of the antibody-incubated column was removed and followed by IP-HPLC. Briefly, each protein sample, 50–100 μg, was mixed with 5 mL of a binding buffer (150 mM NaCl, 10 mM Tris pH 7.4, 1 mM EDTA, 1 mM EGTA, 0.2 mM sodium vanadate, 0.2 mM PMSF and 0.5% NP-40) and incubated in the antibody-bound protein A/G agarose bead column on a rotating stirrer at room temperature for 1 h. After multiple washings of the columns with Tris-NaCl buffer, pH 7.5, in a graded NaCl concentration (0.15–0.3 M), the target proteins were eluted with 300 μL of IgG elution buffer (Pierce, Waltham, MA, USA). The immunoprecipitated proteins were analyzed using a precision HPLC unit (1100 series, Agilent, Santa Clara, CA, USA) equipped with a reverse-phase column and a micro-analytical UV detector system (SG Highteco, Hanam, Republic of Korea). Column elution was performed using 0.15 M NaCl, 20% acetonitrile solution at 0.5 mL/min for 15 min, 30 °C, and the proteins were detected using a UV spectrometer at 280 nm. The control and experimental samples were run sequentially to allow for comparisons. For IP-HPLC analysis, the whole protein peak areas (mAUs*) were obtained and calculated mathematically using an analytical algorithm by subtracting the negative control antibody peak areas, and protein expression levels were compared and normalized using the square roots of the protein peak areas [18].

### 2.7. Micro-Computerized Tomography (μ-CT)

Right femurs were harvested and fixed in 4% PFA at 4 °C for 24 h, and then μ-CT imaging was performed using Quantum FX μ-CT (PerkinElmer, Hopkinton, MA, USA). We obtained the images in the following settings: 9.7 μM voxel resolution, 90 kV and 200 μA, 10 mm field of view, and 3 min exposure time and reconstruction of the serial cross-sectional images using Analyze 12.0 (Overland Park, KS, USA). To analyze the trabecular and cortical bones, we measured the identical regions of interest, a region commencing at 0.7 mm and extending to 2.3 mm from the bottom of the femoral distal growth plate. All bone parameter analyses were measured according to the guidelines of the American Society for Bone and Mineral Research [19]. Trabecular and cortical bone parameters were assessed independently without repeated measures or within-subject correlations.

### 2.8. Statistical Analysis

The value of each group was shown as the mean ± standard deviation. The normality of the data was tested using the Shapiro–Wilk test to confirm the validity of the statistical analyses. The data were subjected to a one-way analysis of variance (ANOVA) to evaluate the differences among the groups. When the ANOVA indicated significant differences, Tukey’s post hoc test was employed to perform pairwise comparisons between the group means. A significant level of *p* < 0.05 was established for all statistical analyses. All analyses were performed using GraphPad Prism software version 10.3.1 (GraphPad Software, Boston, MA, USA).

## 3. Results

### 3.1. Differential Effects of L-Serine and D-Serine on Bone Metabolism

#### 3.1.1. L-Serine Enhances Osteogenic Gene Expression in Osteoblasts

**Figure 1:**
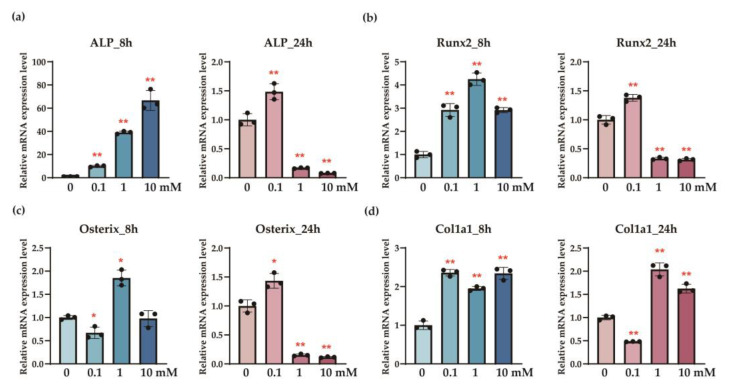
The qRT-PCR analysis through Cq number analysis revealed that the administration of L-serine to MC3T3E1 cells significantly increased the expression levels of *ALP* (**a**), *Runx2* (**b**), *osterix* (**c**), and *Col1a1* (**d**) genes for 8 and 24 h post-administration (* *p* < 0.05, ** *p* < 0.001). Notably, some genes exhibited elevated expression levels up to 24 h after administration. Error bars represent the mean ± standard deviation.

At 8 h post-treatment with L-serine, the relative expression levels of *ALP* mRNA were determined according to the concentration of L-serine (Figure 1). Following treatment with 0.1 mM, 1 mM, and 10 mM L-serine, the expression levels significantly increased compared to the control group, reaching 10.01 ± 0.70, 39.04 ± 1.15, and 66.68 ± 8.60, respectively (*p* < 0.001). Similarly, the relative expression levels of *Runx2* mRNA were significantly elevated in the L-serine-treated groups, with values of 2.92 ± 0.28, 4.25 ± 0.29, and 2.92 ± 0.11 for 0.1 mM, 1 mM, and 10 mM L-serine, respectively (*p* < 0.001). For *osterix* mRNA, a significant increase was observed only in the 10 mM L-serine-treated group, which showed a relative expression level of 1.85 ± 0.17 compared to the untreated control (*p* < 0.05). The expression level of *Col1a1* mRNA also showed significant upregulation across all L-serine treatment concentrations, with values of 2.36 ± 0.08, 1.95 ± 0.05, and 2.34 ± 0.16 for 0.1 mM, 1 mM, and 10 mM L-serine, respectively (*p* < 0.001).

At 24 h post-treatment with L-serine, 0.1 mM L-serine, the expression levels increased significantly to 1.48 ± 0.14 (*p* < 0.001). Similarly, the relative expression levels of *Runx2* mRNA were significantly elevated in the L-serine-treated groups, with values of 1.38 ± 0.06 for 0.1 mM L-serine (*p* < 0.001). For *osterix* mRNA, a significant increase was observed only in the 0.1 mM L-serine-treated group, which showed a relative expression level of 1.43 ± 0.13 compared to the untreated control (*p* < 0.05). The expression levels of *Col1a1* mRNA also showed significant upregulation across all L-serine treatment concentrations, with values of 2.10 ± 0.19 and 1.62 ± 0.09 for 1 mM and 10 mM L-serine, respectively (*p* < 0.001).

#### 3.1.2. D-Serine Suppresses Osteoclast Activation via the Inhibition of Cathepsin K Expression

**Figure 2:**
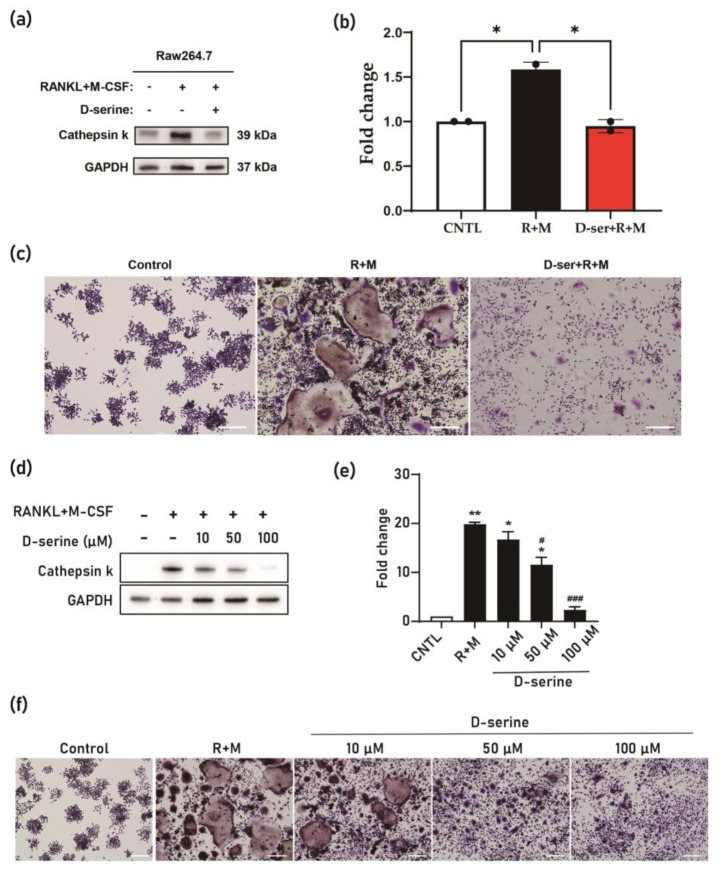
The effect of D-serine administration on osteoclast activation in RAW264.7 cells. (**a**) Treatment with RANKL + M-CSF (R+M) significantly increased the expression of the osteoclast activation marker cathepsin K. Pretreatment with D-serine (50 μM) inhibited R+M-induced osteoclast activation. (**b**) From the results of panel B, compared to the untreated control and D-serine pretreatment groups, the R+M group exhibited significantly higher cathepsin K expression levels (* *p* < 0.05, ** *p* < 0.001). (**c**) Results from the TRAP assay indicated that D-serine pretreatment suppressed osteoclast activation (Scale bar: 200 µm). (**d**,**e**) A concentration-dependent effect of D-serine on cathepsin K expression was observed, with higher concentrations of D-serine resulting in greater inhibition. Compared to the untreated control, R+M, 10 μM D-serine, and 50 μM D-serine treatments significantly increased cathepsin K expression levels (* *p* < 0.05, ** *p* < 0.001). However, relative to the R+M group, 50 μM and 100 μM D-serine treatments significantly reduced cathepsin K expression levels (# *p* < 0.05, ### *p* < 0.001). Error bars represent the mean ± standard deviation. (**f**) Similar trends were observed in the TRAP activity assay, further confirming the dose-dependent inhibitory effect of D-serine on osteoclast activation (Scale bar: 200 µm).

Cathepsin K serves as a key activation marker for osteoclasts and is a therapeutic target in osteoporosis [20]. Treatment with RANKL and M-CSF significantly increased the expression level of cathepsin K in RAW264.7 cells (Figure 2a). Notably, pretreatment with D-serine suppressed RANKL + M-CSF-induced osteoclast activation, as indicated by a decreased expression level of cathepsin K (Figure 2b). This inhibitory effect of D-serine was further substantiated by the TRAP activity assay, which showed reduced TRAP activity following D-serine pretreatment (Figure 2c).

A dose-dependent effect of D-serine was observed, with concentrations ranging from 10 μM to 100 μM progressively reducing RANKL + M-CSF-induced cathepsin K expression (Figure 2d). Significantly, the 50 μM and 100 μM D-serine treatment groups exhibited markedly lower cathepsin K expression levels compared to the RANKL + M-CSF-only group (*p* < 0.05; Figure 2e). Similar trends were observed in the TRAP activity assay, further demonstrating the inhibitory effect of D-serine on osteoclast activation (Figure 2f).

### 3.2. Effects of Sericin on D-Serine Levels and Bone-Related Protein Expression in Blood Serum

**Figure 3:**
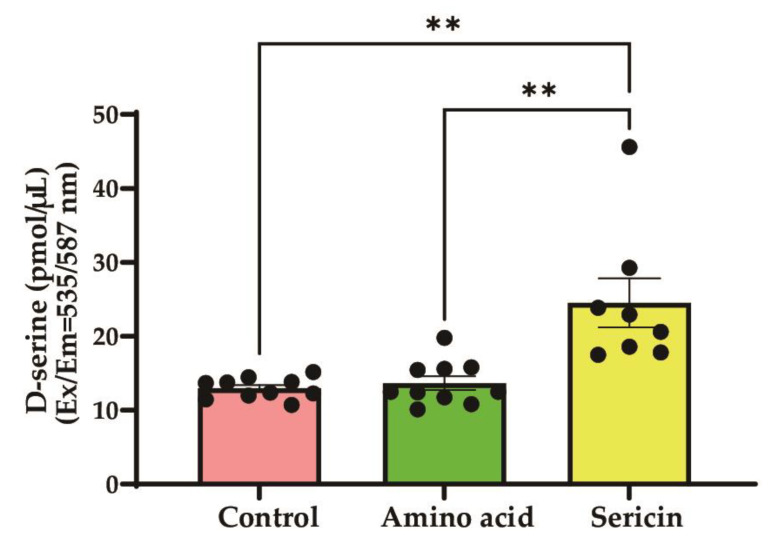
Serum D-serine level in each group. Compared to the control and amino acid mixture groups, the sericin group showed significantly higher serum D-serine level. Error bars represent the mean ± standard deviation (** *p* < 0.001).

The serum D-serine levels were measured in each group (Figure 3). The mean serum D-serine levels were 12.97 ± 1.42 pmol/μL in the control group, 13.67 ± 2.94 pmol/μL in the amino acid mixture group, and 24.52 ± 9.37 pmol/μL in the sericin group. A significant difference was observed among the groups (*p* < 0.001). Post hoc analysis revealed that the serum D-serine level in the sericin group was significantly higher than both the amino acid mixture group (mean difference: 10.85 pmol/μL; 95% CI: 5.80–15.89; *p* < 0.001) and the control group (mean difference: 11.55 pmol/μL; 95% CI: 6.70–16.39; *p* < 0.001).

**Figure 4:**
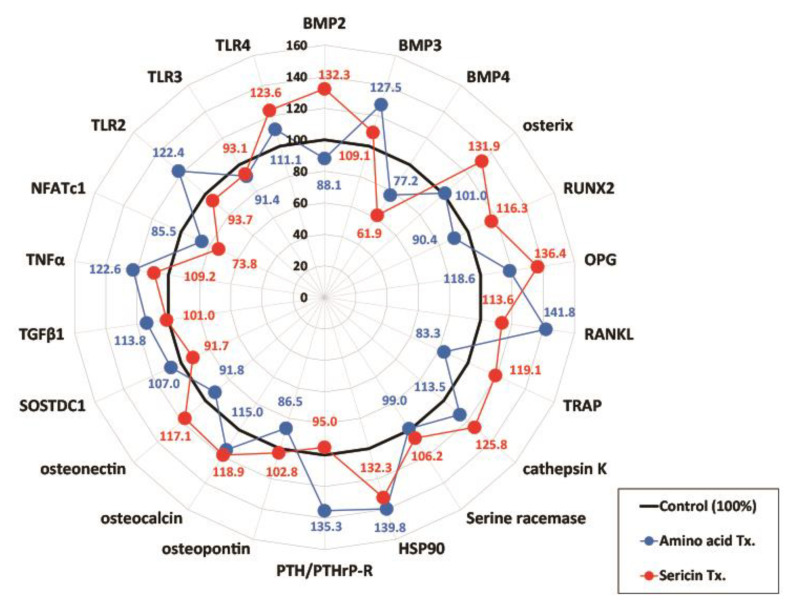
Effects of sericin treatment on serum protein expression. IP-HPLC analysis of serum from the sericin-treated groups showed an increased expression of serine racemase and osteogenic proteins, including BMP2, osterix, Runx2, OPG, osteopontin, osteocalcin, and osteonectin, while reducing anti-osteogenic proteins like BMP3, PTH/PTHrP-R, and SOSTDC1. Osteoclastogenesis was inhibited by downregulating RANKL and HSP90. Inflammation and stromal fibrosis were reduced via downregulation of TNFα, NFATc1, TLR2, TLR3, and TGFβ1. These findings highlight sericin’s dual-action effects on bone metabolism and inflammation, supporting its therapeutic potential for osteoporosis.

In this study, the IP-HPLC analysis of sericin-treated serum revealed elevated serine racemase expression in comparison to the untreated control (Figure 4). The analysis indicated a pronounced osteogenesis-related effect, attributable to the signaling of L-serine and D-serine, which are derived from sericin. The stimulation of osteoblastogenesis was observed, achieved by upregulating the expression of osteogenic proteins, including BMP2, osterix, Runx2, OPG, osteopontin, osteocalcin, and osteonectin, and by downregulating anti-osteogenic proteins, such as BMP3, PTH/PTHrP-R, and SOSTDC1. Conversely, osteoclastogenesis was found to be modulated by (1) the downregulation of the expression of osteoclast differentiation factors, RANKL and HSP90, and (2) the upregulation of the expression of osteoclast activation factors, TRAP and cathepsin K. While TRAP and cathepsin K are typically associated with osteoclast activity, their expression in this context may reflect the role of L-serine in promoting osteoclast differentiation [14], which is essential for coupling with osteoblast activation during bone remodeling. In addition, inflammation was reduced by (1) the reduced expression of pro-inflammatory proteins, TNFα, NFATc1, TLR2, and TLR3, and (2) the downregulation of stromal fibrosis through the downregulation of TGFβ1.

### 3.3. Effects of Sericin Feeding on Trabecular and Cortical Bone in an Osteoporosis Model

**Figure 5:**
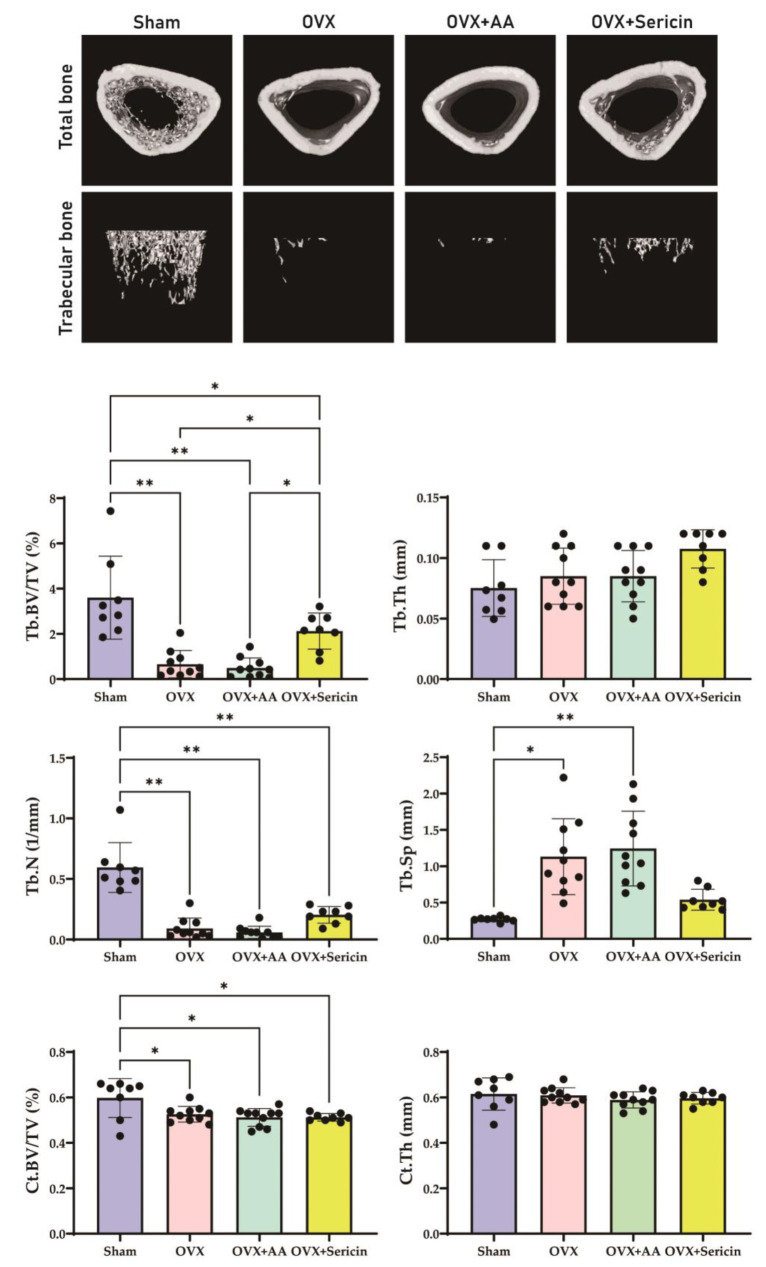
Representative μ-CT images and quantitative analyses of trabecular and cortical bone parameters in the sham, OVX, OVX + AA (amino acid mixture), and OVX + Sericin groups. The trabecular bone volume to tissue volume (Tb.BV/TV) was significantly higher in the OVX + Sericin group compared to the OVX and OVX + AA groups. The trabecular separation (Tb.Sp) was significantly lower in the OVX + Sericin group compared to the OVX and OVX + AA groups, while no significant differences were observed in trabecular number (Tb.N)and trabecular thickness (Tb.Th) between these groups. For cortical bone, no significant differences in cortical bone volume to tissue volume (Ct.BV/TV), cortical thickness (Ct.Th), or cortical area (Ct.Ar) were observed among the OVX groups. Compared to the sham group, the OVX groups exhibited significantly lower Ct.BV/TV and Ct.Ar, with no changes in Ct.Th. The error bars represent the mean ± standard deviation (* *p* < 0.05, ** *p* < 0.001).

Sericin feeding demonstrated the potential to improve bone quality in an osteoporosis animal model (Figure 5). In the μ-CT analysis, the trabecular bone volume to tissue volume (Tb.BV/TV) was significantly higher in the OVX-sericin group (2.124 ± 0.7982) compared to both the OVX group (0.659 ± 0.6062, mean difference: 1.465; 95% CI: 0.321–2.609; *p* = 0.023) and the OVX-amino acid group (0.489 ± 0.4443, mean difference: 1.635; 95% CI: 0.538–2.731; *p* = 0.010). These results demonstrate the substantial effect of sericin treatment on improving trabecular bone quality. However, no significant differences in trabecular number (Tb.N) were observed between the groups. The OVX group, compared to the sham group, exhibited a significant decrease in Tb.BV/TV and Tb.N, an increase in trabecular separation (Tb.Sp), and no notable change in trabecular thickness (Tb.Th). Notably, Tb.Sp was significantly lower in the OVX-sericin group than in the OVX and OVX-amino acid groups (*p* = 0.017 and *p* = 0.004, respectively).

Cortical bone analysis revealed no significant differences in cortical bone volume to tissue volume (Ct.BV/TV), cortical thickness (Ct.Th), or cortical area (Ct.Ar) among the OVX groups (*p* > 0.05). However, compared to the sham group, the OVX groups showed a significant decrease in Ct.BV/TV and Ct.Ar (*p* < 0.05). No significant differences in Ct.Th were observed between the sham group and any of the OVX groups (*p* > 0.05).

## 4. Discussion

This study demonstrates the potential of sericin to modulate bone metabolism in the osteoporosis model through the distinct roles of its components, L-serine and D-serine. L-serine significantly enhanced the expression of osteogenic markers, including ALP, Runx2, osterix, and Col1a1, in osteoblasts (Figure 1), suggesting its role in promoting bone formation. Conversely, D-serine suppressed osteoclast activation by inhibiting cathepsin K expression and reducing TRAP activity in a dose-dependent manner, indicating its anti-resorptive properties (Figure 2). Sericin feeding also increased serum D-serine levels (Figure 3) and upregulated key bone-related proteins, such as BMP-2, osterix, and Runx2 (Figure 4). Additionally, μ-CT analysis revealed improved trabecular bone mass in the OVX-sericin group, evidenced by higher Tb.BV/TV and lower Tb.Sp compared to the OVX and OVX-amino acid groups (Figure 5). These findings collectively highlight the dual mechanism by which sericin enhances bone formation and inhibits bone resorption, offering a promising therapeutic strategy for osteoporosis management.

Sericin, a protein derived from silkworms, is characterized by its unique amino acid composition, particularly its high L-serine content, which constitutes approximately 30% of its structure [21]. This proportion is significantly higher compared to most other proteins, making sericin a potent source of L-serine upon oral administration [12]. In this study, sericin intake led to elevated serum levels of D-serine (Figure 3), a critical regulator of bone metabolism [14]. Approximately 8.55% of the initial L-serine was converted to D-serine by serine racemase within 4 h [17], suggesting that serum levels of L-serine may also be similarly elevated. Previous research has demonstrated sericin’s potential to enhance bone mass in osteoporosis models, likely due to its bioactive amino acid profile [22]. Furthermore, sericin-incorporated grafts have been shown to enhance bone regeneration when applied to bone defects [23]. The findings of this study further underscore sericin’s osteogenic potential, highlighting its dual role in promoting osteoblast activity and mitigating osteoclast activation. These results suggest that sericin could serve as a promising functional food or therapeutic agent for osteoporosis management [24].

The dual roles of L-serine and D-serine in bone metabolism were evident in this study. L-serine significantly enhanced the expression of osteogenic markers, including *ALP, Runx2, osterix*, and *Col1a1*, in osteoblasts, indicating its role in promoting bone formation (Figure 1). Feeding sericin also showed similar changes in the serum proteins (Figure 4). Conversely, D-serine exhibited strong anti-resorptive effects by suppressing osteoclast activation [14]. This was demonstrated through reduced cathepsin K expression and decreased TRAP activity in osteoclast cultures (Figure 2). The conversion of L-serine to D-serine by serine racemase, expressed in osteoblasts, highlights a regulatory mechanism that balances osteogenesis and osteoclastogenesis [14]. These findings emphasize the complementary roles of L-serine and D-serine in maintaining bone homeostasis, which could be leveraged for therapeutic purposes in osteoporosis.

Interestingly, while *ALP* activity demonstrated a clear dose-response relationship with increasing concentrations of L-serine, other osteogenic markers, such as *Runx2*, *osterix*, and *Col1a1*, did not show consistent dose-dependent changes (Figure 1). This discrepancy may reflect the complex nature of osteoblast differentiation, where early markers like ALP [25] are more directly influenced by the metabolic effects of L-serine, whereas later-stage markers might involve additional regulatory mechanisms or signaling pathways. It is also possible that L-serine exerts its effects through a threshold mechanism, where once an optimal concentration is reached, additional increases may not yield proportional changes. Furthermore, saturation of receptor-mediated or metabolic pathways could account for the lack of a linear dose-response for these markers [26]. These findings highlight the need for further studies to elucidate the precise mechanisms underlying L-serine’s actions on osteoblasts, including extending observation periods and evaluating its interaction with other osteogenic pathways.

Current anti-resorptive therapies, such as bisphosphonates and RANKL inhibitors, primarily target the suppression of bone resorption but are often associated with adverse effects, including the risk of MRONJ [27,28]. Sericin, rich in L-serine, offers a unique advantage as L-serine is converted to D-serine, which acts as an osteoclast inhibitor [14] with behavior like that of anti-resorptive drugs. In addition, L-serine enhances osteoblast activity by promoting the expression of osteogenic proteins (Figure 1 and Figure 4). This dual mechanism, simultaneously enhancing osteoblast activity and inhibiting osteoclast differentiation [16], positions sericin as a promising alternative or adjunct to existing osteoporosis therapies. Furthermore, its natural origin and minimal toxicity profile make sericin an attractive candidate for long-term use, particularly in populations at a high risk of osteoporosis [24]. Future clinical studies are needed to validate the efficacy and safety of sericin in comparison to conventional osteoporosis treatments.

**Figure 6:**
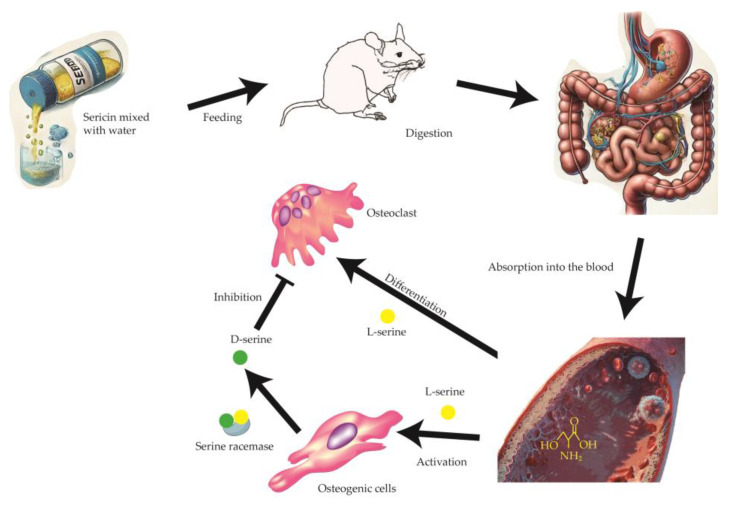
Mechanism of sericin’s effect on bone metabolism. When sericin is fed to rats, it is digested by proteolytic enzymes and absorbed into the bloodstream as amino acids, primarily L-serine. L-serine plays a dual role in bone metabolism: it activates osteogenic cells, promoting bone formation, and facilitates the differentiation of osteoclasts. Differentiated osteoclasts, through coupled bone remodeling reactions, can promote osteoblast activation and enhance bone formation. Additionally, a portion of L-serine is converted into D-serine by the enzyme serine racemase in osteoblasts. D-serine acts as an inhibitor of osteoclast activation, thereby suppressing excessive bone resorption. This dual mechanism of L-serine and D-serine contributes to bone mass increase, particularly under osteoporosis conditions, by maintaining a balance between bone formation and resorption.

The mechanisms underlying sericin’s effects on bone metabolism involve complex interactions between its bioactive components and cellular processes. A critical aspect of these effects is the conversion of L-serine to D-serine by serine racemase [17]. D-serine plays a significant role in inhibiting osteoclast differentiation [14], thereby reducing bone resorption. Additionally, amino acid transporters facilitate the uptake of sericin-derived serine, influencing cellular activities in both osteoblasts and osteoclasts (Figure 6). This interplay highlights the dual role of L-serine in promoting osteoblast activation and osteoclast differentiation while providing the substrate for D-serine-mediated inhibition of osteoclast activity.

Postmenopausal status, characterized by estrogen deficiency, can profoundly influence the synthesis and metabolism of non-essential amino acids [29]. Studies in OVX mice, a widely used model for postmenopausal estrogen deficiency, have shown significant reductions in several plasma amino acid levels [30]. This hormonal shift may impact the availability of amino acids, including L-serine, and consequently affect bone metabolism. Despite these insights, further studies are needed to elucidate the precise signaling pathways through which sericin and its derivatives act. Currently, direct evidence linking serum L-serine levels to osteoporosis in humans is limited. Future research should explore the dose-dependent effects of sericin, its long-term impact on bone quality, and its potential to synergize with existing osteoporosis therapies. Clinical trials will be essential to validate these findings and establish sericin as a viable and effective treatment option for osteoporosis.

While this study demonstrated significant improvements in trabecular bone parameters, including Tb.BV/TV and Tb.Sp, no significant changes were observed in cortical bone parameters, such as Ct.BV/TV, Ct.Th, or Ct.Ar. This discrepancy may be attributed to several factors. First, trabecular bone undergoes more rapid remodeling and is more metabolically active than cortical bone [31,32], making it more responsive to interventions targeting bone metabolism, such as sericin and its components. In contrast, cortical bone, with its slower remodeling rate [33], may require a longer treatment duration or higher dosage to exhibit significant changes. Second, the method of sericin administration may have contributed to the lack of significant effects on cortical bone. As sericin was mixed into the drinking water, the drinking patterns of individual animals could have been irregular. This irregularity may have led to variations in L-serine concentrations in the bloodstream over time. After initial ingestion, the concentration of L-serine may have faded, reducing its impact. This aligns with our osteoblast experiment, where the observed elevation of osteogenic markers at 8 h diminished by 24 h after initial treatment (Figure 1). Regular or more controlled administration, such as daily oral gavage, might ensure a more consistent systemic concentration of L-serine, potentially yielding stronger effects on cortical bone. Finally, sericin’s mechanism of action, primarily through its bioactive components L-serine and D-serine, may favor pathways that are more prominent in trabecular bone remodeling, such as osteoclast-mediated resorption. These findings highlight the need for further investigations to optimize the dosing method, treatment duration, and combination strategies to enhance sericin’s efficacy in cortical bone.

While this study provides valuable insights into the effects of sericin on bone metabolism, it has several limitations. First, the findings are based on an animal model, which may not fully replicate human bone metabolism. Second, the study used a relatively small sample size, which may limit the statistical power and generalizability of the findings. Future studies with larger sample sizes are necessary to confirm the robustness of the observed effects. Third, while this study included untreated and RANKL + M-CSF-treated groups as controls, additional positive controls, such as pharmacological inhibitors of osteoclast differentiation (e.g., bisphosphonates) or activators of osteoblast differentiation (e.g., BMP-2), were not used. Including these controls in future studies would allow for more comprehensive validation of sericin’s efficacy. Fourth, the study did not investigate the variability or potential adverse effects of sericin due to differences in its source or preparation. Fifth, the long-term safety and efficacy of sericin were not assessed, leaving questions about its clinical applicability unanswered. Finally, the interaction between L-serine and D-serine, particularly in the context of their conversion and relative concentrations, warrants further exploration. Addressing these limitations in future studies—by including additional positive controls, validating the results in larger cohorts, assessing long-term outcomes, and exploring the effects of sericin’s source and preparation—will be critical to advancing our understanding of its therapeutic potential.

## 5. Conclusions

This study highlights sericin’s potential as a modulator of bone metabolism, with L-serine enhancing osteoblast activity and D-serine inhibiting osteoclastogenesis. Sericin supplementation improved trabecular bone mass in an osteoporosis model, demonstrating its role in bone remodeling. However, these findings are based on preclinical data, and limitations such as a lack of significant cortical bone effects, a small sample size, and the absence of additional pharmacological controls must be considered. Future research should focus on validating these results in larger cohorts, assessing long-term safety and efficacy, and comparing sericin’s effects with standard osteoporosis treatments. Further mechanistic investigations are also needed to optimize sericin’s therapeutic potential.

## Data Availability

Data are contained within the article.

## Abbreviations

The following abbreviations are used in this manuscript:

μ-CT	micro-computerized tomography
ALP	alkaline phosphatase
ANOVA	analysis of variance
Ar	area
BMD	bone mineral density
BMP	bone morphogenic protein
Col1a1	collagen type I a1
CKLB	Korean cell line bank
Ct.BV/TV	cortical bone volume to tissue volume
DMEM	Dulbecco’s modified eagle medium
FBS	fetal bovine serum
HSP90	heat shock protein 90
IP-HPLC	immunoprecipitation-based high performance liquid chromatography
M-CSF	macrophage colony-stimulating factor
MRONJ	medication-related osteonecrosis of the jaw
NFATc1	nuclear factor of activated T cells, cytoplasmic 1
OPG	osteoprotegerin
OVX	ovariectomy
PTH/PTHrP-R	parathyroid hormone/parathyroid hormone-related peptide receptor
RANKL	receptor activator of nuclear factor kappa-B ligand
Runx2	Runt-related transcription factor 2
SOSTDC1	sclerostin domain containing 1
SPF	specific pathogen-free
TRAP	Tartrate-resistant acid phosphatase
TGFβ1	transforming growth factor-β1
Th	thickness
TLR	toll-like receptor
TNFα	tumor necrosis factor α

## Appendix A

**Table A1:** The primer sequences for qRT-PCR.

Gene	Forward	Reverse
*ALP*	TCC CAC GTT TTC ACA TTC GG	CCC GTT ACC ATA TAG GAT GGC C
*Osx*	AGA GGT TCA CTC GCT CTG ACG A	TTG CTC AAG TGG TCG CTT CTG
*Runx2*	ACA TGG CCA GAT TCA CAG TGG	TGG TGC CCG TTA GCA ATT G
*Col1a1*	CCC AAG GAA AAG AAG CAC GTC	AGG TCA GCT GGA TAG CGA CATC
*GAPDH*	GCA TCT CCC TCA CAA TTT CCA	GTG CAG CGA ACT TTA TTG ATG G

**Table A2:** The list of antibodies used for IP-HPLC.

Name	Catalogue Number	Manufacturer	Location
BMP2	sc-6895	Santa Cruz Biotechnology	Santa Cruz, CA, USA
BMP3	sc-9031	Santa Cruz Biotechnology	Santa Cruz, CA, USA
BMP4	sc-12721	Santa Cruz Biotechnology	Santa Cruz, CA, USA
Osterix	sc-393060	Santa Cruz Biotechnology	Santa Cruz, CA, USA
Runx2	sc-390715	Santa Cruz Biotechnology	Santa Cruz, CA, USA
OPG	sc-11383	Santa Cruz Biotechnology	Santa Cruz, CA, USA
RANKL	sc-7627	Santa Cruz Biotechnology	Santa Cruz, CA, USA
TRAP	sc-376875	Santa Cruz Biotechnology	Santa Cruz, CA, USA
Cathepsin K	sc-48353	Santa Cruz Biotechnology	Santa Cruz, CA, USA
Serine racemase	sc-365217	Santa Cruz Biotechnology	Santa Cruz, CA, USA
HSP70	MA3-007	Invitrogen	Waltham, MA, USA
PTH/PTHrP-R	sc-12722	Santa Cruz Biotechnology	Santa Cruz, CA, USA
Osteopontin	sc-21742	Santa Cruz Biotechnology	Santa Cruz, CA, USA
Osteocalcin	sc-365797	Santa Cruz Biotechnology	Santa Cruz, CA, USA
Osteonectin	sc-25574	Santa Cruz Biotechnology	Santa Cruz, CA, USA
SOSTDC1	sc-162253	Santa Cruz Biotechnology	Santa Cruz, CA, USA
TGFβ1	sc-130348	Santa Cruz Biotechnology	Santa Cruz, CA, USA
TNFα	sc-52746	Santa Cruz Biotechnology	Santa Cruz, CA, USA
NFATc1	sc-17834	Santa Cruz Biotechnology	Santa Cruz, CA, USA
TLR2	sc-21759	Santa Cruz Biotechnology	Santa Cruz, CA, USA
TLR3	sc-32232	Santa Cruz Biotechnology	Santa Cruz, CA, USA
TLR4	sc-293072	Santa Cruz Biotechnology	Santa Cruz, CA, USA
